# Presentation for care and antenatal management of HIV in the UK, 2009−2014

**DOI:** 10.1111/hiv.12410

**Published:** 2016-08-01

**Authors:** CE French, C Thorne, L Byrne, M Cortina‐Borja, PA Tookey

**Affiliations:** ^1^ Population, Policy and Practice ProgrammeUCL Institute of Child HealthUniversity College LondonLondonUK

**Keywords:** antenatal care, antiretroviral therapy, HIV, pregnancy, prevention of mother‐to‐child transmission

## Abstract

**Objectives:**

Despite very low rates of vertical transmission of HIV in the UK overall, rates are higher among women starting antenatal antiretroviral therapy (ART) late. We investigated the timing of key elements of the care of HIV‐positive pregnant women [antenatal care booking, HIV laboratory assessment (CD4 count and HIV viral load) and antenatal ART initiation], to assess whether clinical practice is changing in line with recommendations, and to investigate factors associated with delayed care.

**Methods:**

We used the UK's National Study of HIV in Pregnancy and Childhood for 2009−2014. Data were analysed by fitting logistic regression and Cox proportional hazards models.

**Results:**

A total of 5693 births were reported; 79.5% were in women diagnosed with HIV prior to that pregnancy. Median gestation at antenatal booking was 12.1 weeks [interquartile range (IQR) 10.0–15.6 weeks] and booking was significantly earlier during 2012–2014 *vs*. 2009–2011 (*P* < 0.001), although only in previously diagnosed women. Overall, 42.2% of pregnancies were booked late (≥ 13 gestational weeks). Among women not already on treatment, antenatal ART commenced at a median of 21.4 (IQR18.1–24.5) weeks and started significantly earlier in the most recent time period (*P* < 0.001). Compared with previously diagnosed women, those newly diagnosed during the current pregnancy booked later for antenatal care and started antenatal ART later (both *P* < 0.001). Multivariable analyses revealed demographic variations in access to or uptake of care, with groups including migrants and parous women initiating care later.

**Conclusions:**

Although women are accessing antenatal and HIV care earlier in pregnancy, some continue to face barriers to timely initiation of antenatal care and ART.

## Introduction

Improvements in antiretroviral therapy (ART) and obstetric care have contributed to the lowest ever rate of mother‐to‐child transmission of HIV (MTCT) of 0.5% in the UK and Ireland in recent years 1. However, MTCT rates are higher in some groups of women, such as those starting ART late in pregnancy 1. There are currently around 1200−1300 pregnancies to diagnosed HIV‐positive women in the UK annually 2; optimizing their care is important not only to prevent transmission of HIV to their children, but also to ensure good maternal health, and the best possible pregnancy outcome.

The antenatal ‘booking’ appointment is generally the first contact pregnant women have with maternity services, and provides an opportunity for the identification of maternal and familial risk factors, the offer of timely antenatal screening, and an opportunity to communicate important information to women 3. Early booking is particularly important for women living with HIV, whether diagnosed before or during the pregnancy, to ensure that they receive the HIV‐specific care they require in a timely manner, including assessment of clinical and virological status, and initiation of ART for those not already on treatment.

Current international and UK guidelines recommend earlier initiation of antenatal ART than previously 4, 5. Identifying population groups at increased risk of late antenatal booking and ART initiation will help target efforts towards ensuring equity in access to and uptake of care. Our objective was to investigate the timing of key elements of care of HIV‐positive pregnant women in the UK [antenatal care booking, HIV laboratory assessment (CD4 count and HIV viral load) and antenatal ART initiation], to assess whether clinical practice has changed in line with the recommendations, and to investigate factors associated with delayed antenatal booking and initiation of antenatal ART.

## Methods

### Data source

The National Study of HIV in Pregnancy and Childhood (NSHPC) is a well‐established, comprehensive, active surveillance study covering the UK and Ireland. Demographic, clinical and obstetric information is collected using standardized reporting forms 6.

### Definitions and classifications

Data on UK pregnancies only, resulting in a live or still birth delivered from 1 January 2009 onwards, and reported by the end of June 2014, were utilized. Some degree of reporting delay is to be expected for pregnancies occurring in more recent years. Pregnancies without information on the timing of HIV diagnosis were excluded (*n* = 10). Two calendar periods, 2009–2011 and 2012–2014, were compared to examine changes over time; the earlier period was covered by the 2008 British guidelines recommending antenatal ART initiation by 28 gestational weeks 7, and the second by 2012 guidelines recommending ART initiation by 24 weeks 4. Both guidelines recommended earlier initiation in women requiring treatment for their own health, and those with high viral loads. We defined late antenatal care booking as booking at ≥ 13 completed gestational weeks, in line with general pregnancy guidelines 3, 8. Analyses were stratified by the timing of HIV diagnosis: pregnancies in previously diagnosed women (further stratified according to ART status at conception where appropriate) and those in women diagnosed after conception (‘newly diagnosed’).

Assessment of the timing of the earliest laboratory test was restricted to newly diagnosed women and based on reported date of earliest CD4 count or viral load test (restricted to those taken between 14 days prior to conception and 7 days after delivery). Baseline (pre‐ART) measurements were further restricted to those prior to or up to 7 days after ART initiation (≤ 7 days after delivery in women receiving no antenatal ART). Viral load measurements were grouped reflecting cut‐offs for recommended timing of ART initiation in women not requiring treatment for their own health 4. Analyses of timing of antenatal ART initiation were, by definition, restricted to pregnancies not conceived on treatment.

### Statistical methods

Data were analysed using stata 12.1 (Stata Corp., College Station, TX). Proportions were calculated among those with known information on the variable of interest and compared using the *χ* ^2^ test or *χ* ^2^ test for trend. Medians are presented with interquartile range (IQR) and were compared using the Wilcoxon−Mann−Whitney test.

Logistic regression models were fitted to identify factors associated with late antenatal care booking. Kaplan–Meier analysis was used to describe time to ART initiation graphically, and factors associated with later antenatal ART initiation were analysed using Cox proportional hazards modelling. The proportional hazards assumption was assessed by examining log‐log plots and the model's Schoenfeld residuals 9. The Therneau−Grambsch test was significant both globally and locally for time period, CD4 count and viral load in both strata; this is not surprising given the relatively large sample sizes. There was evidence of nonparallelism and crossings in the log‐log plots; however, these were mainly in the lower and upper tails of the distribution of time to ART initiation (and thus related to relatively small numbers of women), while the central part of this distribution showed little evidence of departures from the assumption. Variables with overall significance at the *P* < 0.1 level in univariable analyses were included in multivariable models, with other variables only included if they improved the fit (assessed using the Wald test). Time period was included *a priori*. Some women with a prior diagnosis contributed more than one pregnancy to these analyses, and this clustering was accounted for by using robust standard errors 10.

### Ethical approval

The National Study of HIV in Pregnancy and Childhood has London Multi‐Centre Research Ethics Committee approval (MREC/04/2/009).

## Results

### Study population

By the end of June 2014, 5693 eligible pregnancies had been reported (5641 live and 52 still births) in 4962 women (3506 deliveries in 2009–2011 and 2187 in 2012–2014). Overall, 2886 (50.7%) pregnancies were among women conceiving on ART, 1601 (28.1%) among diagnosed women not on ART at conception and 1166 (20.5%) among women newly diagnosed during the current pregnancy; the remaining 40 pregnancies were to women diagnosed before conception, but missing information on ART status at conception (Table 1). The proportion of pregnancies in women with previously diagnosed HIV increased from 76.6% in 2009–2011 to 84.2% in 2012–2014 (*P* < 0.001). Overall, most pregnancies were in women from sub‐Saharan Africa (75.3%), over two‐fifths were reported in London, nearly three‐quarters were in parous women, and half were conceived on ART.

**Table 1:** Pregnancy characteristics stratified by timing of HIV diagnosis

Characteristic	Diagnosed prior to current pregnancy ‐ not on ART at conception	Diagnosed prior to current pregnancy ‐ on ART at conception	Diagnosed prior to current pregnancy (irrespective of ART status)^a^	Newly diagnosed during current pregnancy	Total (all pregnancies)
Total	1601	2886	4527	1166	5693 (100)
Time period					
2009–2011	1049 (65.5)	1611 (55.8)	2686 (59.3)	820 (70.3)	3506 (61.6)
2012–2014	552 (34.5)	1275 (44.2)	1841 (40.7)	346 (29.7)	2187 (38.4)
Maternal age at delivery					
< 25 years	209 (13.1)	140 (4.9)	355 (7.8)	177 (15.2)	532 (9.3)
25–34 years	967 (60.4)	1479 (51.2)	2472 (54.6)	727 (62.3)	3199 (56.2)
≥ 35 years	425 (26.5)	1267 (43.9)	1700 (37.6)	262 (22.5)	1962 (34.5)
Maternal region of origin					
UK/Ireland	276 (17.4)	331 (11.6)	612 (13.7)	157 (13.7)	769 (13.7)
Europe	80 (5.0)	107 (3.8)	190 (4.2)	77 (6.7)	267 (4.8)
Eastern Africa	642 (40.5)	1386 (48.7)	2048 (45.8)	397 (34.7)	2445 (43.5)
Middle Africa	100 (6.3)	190 (6.7)	293 (6.6)	49 (4.3)	342 (6.1)
Western Africa	311 (19.6)	498 (17.5)	813 (18.2)	301 (26.3)	1114 (19.8)
Southern Africa	77 (4.9)	130 (4.6)	209 (4.7)	61 (5.3)	270 (4.8)
Africa (unspecified)	11 (0.7)	30 (1.1)	41 (0.9)	18 (1.6)	59 (1.1)
Elsewhere	88 (5.6)	174 (6.1)	265 (5.9)	85 (7.4)	350 (6.2)
Reporting region					
London	641 (40.2)	1279 (44.4)	1926 (42.6)	488 (42)	2414 (42.5)
Midlands and the East	409 (25.6)	649 (22.5)	1073 (23.8)	284 (24.4)	1357 (23.9)
North	253 (15.9)	414 (14.4)	677 (15)	153 (13.2)	830 (14.6)
South	189 (11.8)	394 (13.7)	591 (13.1)	150 (12.9)	741 (13.1)
Wales, Scotland and Northern Ireland	103 (6.5)	145 (5)	249 (5.5)	87 (7.5)	336 (5.9)
Parity					
Nulliparous	300 (19.6)	559 (20.4)	864 (20.1)	555 (50.7)	1419 (26.3)
One previous birth	592 (38.7)	1018 (37.2)	1626 (37.9)	323 (29.5)	1949 (36.2)
Two previous births	400 (26.1)	740 (27.0)	1145 (26.7)	145 (13.2)	1290 (23.9)
At least three previous births	238 (15.6)	419 (15.3)	660 (15.4)	72 (6.6)	732 (13.6)
Earliest CD4 count^b^					
≥ 500 cells/μL	484 (33.1)	1163 (44.2)	1654 (40.3)	306 (27.9)	1960 (37.7)
350–499 cells/μL	432 (29.5)	800 (30.4)	1238 (30.2)	258 (23.6)	1496 (28.8)
200–349 cells/μL	368 (25.2)	517 (19.7)	886 (21.6)	317 (28.9)	1203 (23.1)
< 200 cells/μL	178 (12.2)	149 (5.7)	327 (8.0)	214 (19.5)	541 (10.4)
Earliest viral load^b^					
< 50 copies/mL	275 (18.1)	2350 (85.9)	2641 (61.8)	145 (12.8)	2770 (51.4)
50–29 999 copies/mL	931 (61.4)	342 (12.5)	1279 (29.9)	700 (61.6)	1973 (36.6)
30 000–99 999 copies/mL	213 (14.0)	25 (0.9)	238 (5.6)	168 (14.8)	406 (7.5)
≥ 100 000 copies/mL	98 (6.5)	18 (0.7)	116 (2.7)	124 (10.9)	240 (4.5)

ART, antiretroviral therapy.

Values are *n* (%).

a Group combines the first two groups presented in the table (*n* = 1601 + 2886) plus 40 pregnancies with missing information on ART status at conception.

b Earliest antenatal measurement in that pregnancy, *not* restricted to measurements taken prior to ART initiation.

### Timing of booking for antenatal care

**Figure 1:**
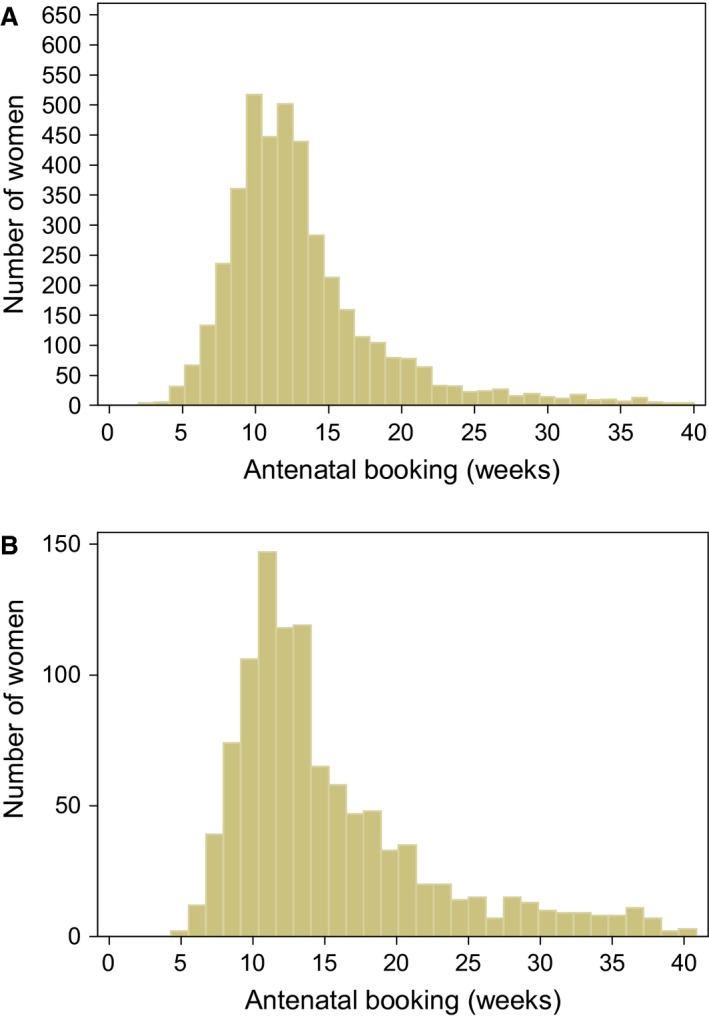
Gestation at antenatal care booking for (a) women diagnosed with HIV prior to that pregnancy and (b) women newly diagnosed with HIV during that pregnancy.

Booking date was reported for 5162 (90.7%) pregnancies. It was less well reported for pregnancies from London compared with the rest of England (*P* < 0.001), and better reported for pregnancies in parous women (*P* < 0.001). Gestational age at booking had a right‐skewed distribution, with a median (IQR) of 12.1 (10.0–15.6) weeks. Overall, 42.2% of pregnancies were booked late at ≥ 13 completed weeks: 25.7% at 13–17 weeks, 10.0% at 18–23 weeks, and 6.6% at ≥ 24 weeks. The proportion of pregnancies booked late declined from 44.8% in 2009–2011 to 38.2% in 2012–2014 (*P* < 0.001), although only in previously diagnosed women. Pregnancies in previously diagnosed women were booked earlier than those in newly diagnosed women at a median of 12.0 (9.7–14.9) weeks compared with 13.1 (10.6–18.0) weeks (*P* < 0.001), respectively (Fig. 1a,b); 39.7% of pregnancies in previously diagnosed women were booked late at ≥ 13 weeks (including 14.2% at ≥ 18 weeks), compared with 51.9% (25.7% at ≥ 18 weeks) in newly diagnosed women (both *P* < 0.001). In previously diagnosed women, there was no association between gestational age at booking and CD4 count (*P* = 0.33).

In univariable analyses, maternal region of origin, UK geographical area of report, and parity were significantly associated with late booking in pregnancies to both previously and newly diagnosed women (Table 2). Additionally, in pregnancies to previously diagnosed women, earlier time period, low antenatal CD4 count and not conceiving on ART were associated with increased odds of late booking.

**Table 2:** Univariable and multivariable analyses of factors associated with booking late for antenatal care (≥ 13 gestational weeks), stratified by timing of HIV diagnosis

	Diagnosed prior to current pregnancy					Newly diagnosed during current pregnancy				
	Booked at ≥ 13 weeks/total (%)	OR (95% CI)	*P*‐value	aOR (95% CI)^a^	*P*‐value	Booked at ≥ 13 weeks/total (%)	OR (95% CI)	*P*‐value	aOR (95% CI)^b^	*P*‐value
Time period of delivery										
2009–2011	1027/2415 (42.5)	1				390/748 (52.1)	1		1	
2012–2014	600/1681 (35.7)	0.75 (0.66–0.85)	< 0.001	0.74 (0.64–0.86)	< 0.001	163/318 (51.3)	0.97 (0.74–1.26)	0.792	1.00 (0.74–1.34)	0.978
Maternal age at delivery										
< 25 years	126/318 (39.6)	1				84/162 (51.9)	1			
25–34 years	855/2232 (38.3)	0.95 (0.74–1.21)	0.654	–		343/666 (51.5)	0.99 (0.70–1.39)	0.936	–	
≥ 35 years	646/1546 (41.8)	1.09 (0.85–1.40)	0.481	–	–	126/238 (52.9)	1.04 (0.70–1.56)	0.830	–	–
Maternal region of origin										
UK/Ireland	177/551 (32.1)	1		1		48/140 (34.3)	1		1	
Europe	64/173 (37.0)	1.24 (0.86–1.79)	0.249	1.51 (1.01–2.26)	0.044	31/70 (44.3)	1.52 (0.85–2.74)	0.160	1.54 (0.81–2.95)	0.188
Eastern Africa	758/1823 (41.6)	1.50 (1.22–1.85)	< 0.001	1.61 (1.28–2.02)	< 0.001	197/351 (56.1)	2.45 (1.63–3.69)	< 0.001	2.73 (1.74–4.29)	< 0.001
Middle Africa	130/264 (49.2)	2.05 (1.50–2.81)	< 0.001	2.24 (1.59–3.14)	< 0.001	33/48 (68.8)	4.22 (2.09–8.52)	< 0.001	3.84 (1.82–8.13)	< 0.001
Western Africa	304/763 (39.8)	1.40 (1.10–1.77)	0.006	1.65 (1.27–2.14)	< 0.001	159/284 (56)	2.44 (1.60–3.71)	< 0.001	2.14 (1.33–3.43)	0.002
Southern Africa	81/190 (42.6)	1.57 (1.11–2.23)	0.012	1.65 (1.13–2.43)	0.010	39/58 (67.2)	3.93 (2.05–7.54)	< 0.001	3.92 (1.90–8.05)	< 0.001
Africa (region unspecified)	14/37 (37.8)	1.29 (0.63–2.64)	0.492	1.46 (0.68–3.16)	0.335	7/18 (38.9)	1.22 (0.44–3.35)	0.700	1.52 (0.49–4.73)	0.467
Elsewhere	84/248 (33.9)	1.08 (0.78–1.51)	0.639	1.26 (0.88–1.81)	0.207	30/78 (38.5)	1.20 (0.67–2.13)	0.538	1.25 (0.67–2.33)	0.490
Reporting region										
London	656/1852 (35.4)	1		1		265/459 (57.7)	1		1	
Midlands and the East	353/836 (42.2)	1.33 (1.13–1.58)	0.001	1.42 (1.17–1.71)	< 0.001	118/240 (49.2)	0.71 (0.52–0.97)	0.031	0.65 (0.45–0.93)	0.021
North	285/651 (43.8)	1.42 (1.18–1.71)	< 0.001	1.36 (1.10–1.67)	0.004	58/148 (39.2)	0.47 (0.32–0.69)	< 0.001	0.39 (0.25–0.59)	< 0.001
South	212/512 (41.4)	1.29 (1.05–1.58)	0.015	1.53 (1.22–1.93)	< 0.001	64/133 (48.1)	0.68 (0.46–1.00)	0.050	0.66 (0.42–1.03)	0.069
Wales, Scotland and Northern Ireland	119/237 (50.2)	1.84 (1.40–2.42)	< 0.001	2.06 (1.50–2.84)	< 0.001	46/83 (55.4)	0.91 (0.57–1.46)	0.695	1.18 (0.69–2.01)	0.545
Parity										
Nulliparous	254/799 (31.8)	1		1		265/525 (50.5)	1		1	
One previous birth	541/1459 (37.1)	1.26 (1.05–1.52)	0.012	1.19 (0.97–1.45)	0.091	150/293 (51.2)	1.03 (0.77–1.37)	0.844	1.05 (0.77–1.44)	0.742
Two previous births	460/1033 (44.5)	1.72 (1.42–2.09)	< 0.001	1.60 (1.30–1.98)	< 0.001	78/126 (61.9)	1.59 (1.07–2.37)	0.022	1.87 (1.21–2.87)	0.004
At least three previous births	297/605 (49.1)	2.07 (1.66–2.58)	< 0.001	1.92 (1.52–2.44)	< 0.001	36/64 (56.3)	1.26 (0.75–2.13)	0.384	1.29 (0.73–2.29)	0.377
On ART at conception										
No	650/1458 (44.6)	1		1		n/a	–		n/a	
Yes	964/2602 (37.0)	0.73 (0.64–0.84)	< 0.001	0.70 (0.61–0.82)	< 0.001	n/a	–	–	n/a	–
Earliest CD4 count^c^										
≥ 500 cells/μL	557/1492 (37.3)	1		1		138/278 (49.6)	1		1	
350–499 cells/μL	460/1125 (40.9)	1.16 (0.99–1.36)	0.067	1.12 (0.95–1.33)	0.183	120/235 (51.1)	1.06 (0.75–1.50)	0.748	(dropped)	–
200–349 cells/μL	330/796 (41.5)	1.19 (1.00–1.42)	0.057	1.10 (0.91–1.33)	0.331	153/292 (52.4)	1.12 (0.80–1.55)	0.510	0.93 (0.64–1.36)	0.706
< 200 cells/μL	152/300 (50.7)	1.72 (1.34–2.22)	< 0.001	1.67 (1.27–2.18)	< 0.001	105/196 (53.6)	1.17 (0.81–1.69)	0.399	0.95 (0.66–1.36)	0.781
Earliest viral load^c^										
< 30 000 copies/mL	1416/3552 (39.9)	1				402/775 (51.9)	1			
30 000–99 999 cells/μL	101/213 (47.4)	1.36 (1.02–1.81)	0.034	–		81/156 (51.9)	1.00 (0.71–1.41)	0.991	–	
≥ 100 000 cells/μL	45/106 (42.5)	1.11 (0.74–1.67)	0.606	–	–	57/111 (51.4)	0.98 (0.66–1.46)	0.918	–	–

aOR, adjusted odds ratio; ART, antiretroviral therapy; CI, confidence interval; OR, odds ratio.

a The multivariable model for pregnancies in previously diagnosed women included 3493 observations (and analyses were adjusted for 3149 clusters; i.e. the 3493 pregnancies occurred in 3149 women).

b The multivariable model for pregnancies in women diagnosed during their current pregnancy included 938 observations.

c Earliest antenatal measurement in that pregnancy, *not* restricted to measurements prior to ART initiation.

Both multivariable models included all variables from the univariable analyses, except maternal age and viral load, which did not improve the fit (Table 2). In multivariable analyses of pregnancies in previously diagnosed women, late booking was associated with earlier time period, originating from abroad (including European‐born women and those from all regions of sub‐Saharan Africa), pregnancy reported from outside London, being parous, not conceiving on ART, and having a low CD4 count. Among pregnancies to newly diagnosed women, late booking was associated with sub‐Saharan African origin (all regions), and higher parity. The odds of late booking were lower for pregnancies reported from elsewhere in England compared with London.

In a sensitivity analysis of the association between being born abroad and late antenatal booking which excluded women known to have arrived in the UK at ≥ 13 gestational weeks (*n* = 86), the results were little changed and the strong association between African origin and late booking, regardless of timing of diagnosis, remained (data not shown).

### Timing of first reported laboratory test (CD4 count or viral load) in pregnancies among newly diagnosed women

The timing of the first reported antenatal HIV‐related laboratory test was available for 1139 of 1166 (97.7%) pregnancies. It was at a median of 17.0 weeks of gestation (IQR 13.4–23.4 weeks), and the interval between conception and testing decreased between the two time periods [median 17.4 (IQR 13.7–23.6) weeks in 2009–2011 and 16.0 (IQR 12.9–22.6) weeks in 2012–2014; *P* = 0.016]. Information on both antenatal booking date and date of first antenatal laboratory test was available for 1044 pregnancies (89.5%). The laboratory test was on or after the booking date in 908 of 1044 pregnancies; among these, the median time from booking to laboratory assessment was 2.9 weeks (IQR 1.3–5.9 weeks), with over a third (343 of 908) having a lag of ≥ 4 weeks.

### Timing of antenatal ART initiation

**Figure 2:**
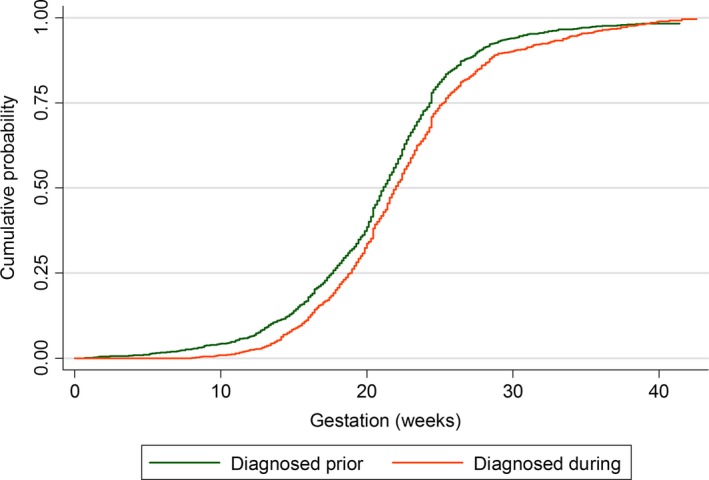
Cumulative probability of initiating antenatal antiretroviral therapy (ART) by gestational week, stratified by timing of HIV diagnosis.

In almost all pregnancies, women received antenatal ART (99.2%; 5620 of 5666), and in most this was combination ART (cART) (98.3%; 5507 of 5602; unspecified regimen in 18). Just over half of all pregnancies were conceived on treatment (*n* = 2886), and virtually all women not on ART at conception started ART during pregnancy (98.3%; 2704 of 2750); start date was available for 2619 (1543 with prior diagnosis). Median gestation at ART initiation among all those initiating ART in pregnancy was 21.4 (IQR 18.1–24.5) weeks [21.0 (IQR 17.6–24.1) weeks in previously diagnosed women, compared with 21.9 (IQR 18.7–25.0) weeks for newly diagnosed women; *P* < 0.001] (Fig. 2). ART was started significantly earlier in pregnancies delivered in 2012–2014 [median 20.3 (IQR 16.9–23.4) weeks] compared with 2009–2011 [median 22.0 (IQR 18.9–24.7) weeks; *P* < 0.001], a trend apparent for both previously and newly diagnosed women (both *P* < 0.001).

Among women not on ART at conception, the median time from antenatal booking to ART initiation was 7.1 (IQR 2.9–11.1) weeks; 7.3 (IQR 2.6–11.6) weeks in those previously diagnosed and 6.9 (IQR 3.0–10.9) weeks in those newly diagnosed. Among pregnancies to newly diagnosed women, the median gap from laboratory testing to ART initiation was 3.0 (IQR 0.3–6.6) weeks. The time from laboratory testing to ART initiation was shorter for pregnancies delivered in 2012–2014 than for those delivered in 2009–2011 [median 2.7 (IQR 0.1–6.0) *vs*. 3.0 (IQR 0.4–7.0) weeks, respectively; *P* = 0.03]; it was also shorter for those with a pre‐ART antenatal CD4 count of < 350 *vs*. ≥ 350 cells/μL [median 2.7 (IQR 1.1–5.0) *vs*. 5.1 (IQR 2.0–9.1) weeks, respectively; *P* < 0.001], and those with viral loads ≥ 30 000 HIV‐1 RNA copies/mL [median 2.3 (IQR 0.9–4.7) weeks] compared with < 30 000 copies/mL [median 4.3 (IQR 1.7–8.1) weeks] (*P* < 0.001).

In univariable analyses of factors associated with the timing of antenatal ART initiation (Table 3), ART was started significantly earlier for pregnancies delivered in the later period compared with 2009–2011, regardless of the timing of HIV diagnosis. Factors associated with shorter time to ART initiation among previously diagnosed women were being reported outside London, having a lower CD4 count and having a higher viral load. There was a similar pattern for newly diagnosed women, but additionally, there were differences according to maternal age and world region of origin, with older women starting ART earlier, and women from Western Africa, in particular, starting later than UK/Irish‐born women.

**Table 3:** Univariable and multivariable Cox proportional hazards analyses of factors associated with shorter time to antenatal antiretroviral therapy (ART) initiation, stratified by timing of HIV diagnosis

Characteristic	Diagnosed prior to current pregnancy					Newly diagnosed during current pregnancy				
	*n*	HR (95% CI)	*P*‐value	aHR (95% CI)^a^	*P*‐value	*n*	HR (95% CI)	*P*‐value	aHR (95% CI)^b^	*P*‐value
Time period										
2009–2011	1022	1		1		796	1		1	
2012–2014	521	1.24 (1.10–1.39)	< 0.001	1.28 (1.12–1.46)	< 0.001	326	1.20 (1.05–1.37)	0.006	1.34 (1.15–1.55)	< 0.001
Maternal age at delivery										
< 25 years	203	1				173	1		1	
25–34 years	932	0.98 (0.83–1.16)	0.800	–		701	1.12 (0.95–1.33)	0.185	1.15 (0.95–1.40)	0.159
≥ 35 years	408	0.97 (0.81–1.16)	0.734	–	–	248	1.31 (1.08–1.60)	0.007	1.17 (0.93–1.47)	0.192
Maternal region of origin										
UK/Ireland	270	1		–		154	1		1	
Europe	78	1.09 (0.85–1.41)	0.483	–	–	71	0.87 (0.66–1.16)	0.357	0.75 (0.55–1.02)	0.068
Eastern Africa	616	1.05 (0.90–1.23)	0.520	–	–	385	0.93 (0.77–1.13)	0.473	0.75 (0.60–0.93)	0.010
Middle Africa	96	0.96 (0.73–1.26)	0.784	–	–	45	0.91 (0.65–1.27)	0.591	0.82 (0.56–1.20)	0.309
Western Africa	303	0.93 (0.78–1.11)	0.436	–	–	294	0.73 (0.60–0.89)	0.002	0.60 (0.48–0.76)	< 0.001
Southern Africa	74	1.08 (0.86–1.35)	0.499	–	–	58	0.77 (0.56–1.04)	0.090	0.68 (0.47–0.97)	0.032
Africa (region unspecified)	10	1.01 (0.68–1.51)	0.951	–	–	15	0.98 (0.58–1.67)	0.939	0.50 (0.25–1.00)	0.049
Elsewhere	83	1.02 (0.80–1.31)	0.881	–	–	81	1.17 (0.89–1.54)	0.249	0.92 (0.68–1.25)	0.612
Reporting region										
London	621	1		1		473	1		1	
Midlands and the East	394	1.17 (1.02–1.33)	0.021	1.13 (0.96–1.32)	0.131	265	1.16 (1.00–1.35)	0.054	1.12 (0.93–1.35)	0.236
North	240	1.28 (1.10–1.48)	0.001	1.31 (1.10–1.57)	0.003	153	1.28 (1.07–1.54)	0.008	1.25 (1.01–1.55)	0.039
South	183	1.11 (0.94–1.31)	0.219	1.00 (0.82–1.22)	0.994	144	1.22 (1.01–1.47)	0.038	1.19 (0.94–1.50)	0.140
Wales, Scotland and Northern Ireland	99	1.21 (0.98–1.50)	0.079	1.32 (1.02–1.71)	0.035	84	1.24 (0.98–1.57)	0.068	1.20 (0.93–1.55)	0.165
Parity										
Nulliparous	289	1		1		538	1		–	
One previous birth	569	0.89 (0.77–1.03)	0.120	0.98 (0.82–1.16)	0.782	312	1.00 (0.87–1.15)	0.971	–	–
Two previous births	389	0.90 (0.77–1.05)	0.192	0.94 (0.79–1.13)	0.525	141	1.04 (0.86–1.25)	0.682	–	–
At least three previous births	226	0.72 (0.59–0.87)	0.001	0.77 (0.63–0.95)	0.013	68	0.98 (0.76–1.26)	0.852	–	–
Baseline CD4 count^c^										
≥ 500 cells/μL	388	1		1		253	1		1	
350–499 cells/μL	341	1.15 (1.01–1.30)	0.036	1.13 (0.97–1.32)	0.109	225	1.04 (0.87–1.25)	0.647	1.02 (0.84–1.23)	0.871
200–349 cells/μL	288	1.38 (1.17–1.63)	< 0.001	1.32 (1.12–1.55)	0.001	280	1.24 (1.04–1.47)	0.015	1.31 (1.09–1.58)	0.003
< 200 cells/μL	144	1.30 (1.02–1.65)	0.032	1.16 (0.93–1.45)	0.199	191	1.54 (1.28–1.86)	< 0.001	1.53 (1.23–1.91)	< 0.001
Baseline viral load^c^, ^d^										
< 30 000 copies/mL	899	1		1		679	1		1	
30 000–99 999 copies/mL	207	1.27 (1.09–1.49)	0.002	1.24 (1.05–1.46)	0.013	159	1.14 (0.96–1.36)	0.130	1.01 (0.83–1.23)	0.904
≥ 100 000 copies/mL	90	1.56 (1.13–2.16)	0.007	1.43 (1.11–1.85)	0.006	119	1.32 (1.08–1.60)	0.006	1.21 (0.96–1.51)	0.103

aHR, adjusted hazard ratio; CI, confidence interval; HR, hazard ratio.

An HR of > 1 indicates a shorter time to ART initiation.

a The multivariable model for pregnancies in previously diagnosed women included 1074 observations (and analyses were adjusted for 996 clusters; i.e. the 1074 pregnancies occurred in 996 women).

b The multivariable model for pregnancies in women diagnosed during their current pregnancy included 903 observations.

c First antenatal measurement in that pregnancy, restricted to measurements prior to ART initiation.

d Viral load grouping based on cut‐offs used for recommending timing of ART initiation in women not requiring treatment for their own health [4.]

In the multivariable model for pregnancies in previously diagnosed women (Table 3), later time period remained associated with earlier ART initiation (*P* < 0.001). Compared with London, ART was started somewhat earlier in the North of England (*P* = 0.003) and in Wales, Scotland and Northern Ireland (*P* = 0.035), and women with at least three previous births started later than nulliparous women (*P* = 0.013). There was some suggestion that those with lower CD4 counts started earlier than those with counts ≥ 500 cells/μL, but this only reached statistical significance for those with counts of 200–350 cells/μL (*P* = 0.001). Those with viral loads > 30 000 copies/mL started earlier than those with lower viral loads (*P* < 0.01). There was no evidence that including maternal age or world region of origin improved the fit of the model. In a sensitivity analysis excluding women known to have arrived in the UK after conception (*n* = 10), the results were little altered.

For pregnancies in newly diagnosed women, the pattern was similar, with ART started earlier in the later period (*P* < 0.001). Women from Eastern, Western, and Southern Africa started later than those from the UK/Ireland (all *P* < 0.05), with some evidence that women from Europe did too (*P* = 0.068). ART was started earlier in all regions/areas of the UK compared with London, although this was only significant for the North of England (*P* = 0.039). Lower CD4 counts were associated with earlier ART. When pregnancies to women known to have arrived in the UK after conception were excluded (*n* = 54), similar patterns were observed, although there was less evidence of an association between Eastern or Southern African origin and later ART (*P* = 0.095 and *P* = 0.081, respectively), while women from Western Africa remained more likely to start ART later (*P* = 0.004) (data not shown). Finally, when the model was restricted to women who booked at < 13 weeks, evidence remained that women born abroad initiated ART later than those born in the UK/Ireland. In particular, women from Western Africa started later [adjusted hazard ratio (aHR) 0.50; 95% confidence interval (CI) 0.34–0.74; *P* < 0.001], as did those from Southern Africa (*P* = 0.05).

### Infant HIV status

HIV status was reported for 3988 infants by the time of this analysis – 16 were perinatally infected (half of whom were born to women with a prior diagnosis). Three‐quarters of perinatally infected infants (*n* = 12) were born to women who booked late, 11 of whom were not on ART at conception.

## Discussion

During the study period, the vast majority (99%) of women received antenatal ART, median gestation at antenatal ART initiation declined over time, and women with high viral loads started ART earlier, in line with changes to UK recommendations 4. Longer duration of antenatal ART is associated with a lower risk of vertical transmission of HIV, with a rapid decline in maternal viral load during the first few weeks of ART 1, 11. The crude vertical transmission rate for deliveries during 2009–2014 is consistent with the previously published UK rate of 0.46% for 2010/2011 1. Although there was a trend towards earlier booking for antenatal care (among previously but not newly diagnosed women), over two‐fifths of pregnancies in HIV‐positive women were booked at ≥ 13 weeks (38% for 2012–2014), compared with 28% of pregnancies in England overall during 2012–2013 12. Later booking among HIV‐positive women probably reflects the cultural and sociodemographic characteristics of this population, and these analyses revealed some important demographic variations in the timeliness of antenatal booking and antenatal ART initiation.

Women from sub‐Saharan Africa were more likely to book late, irrespective of the timing of diagnosis; being born outside the UK and/or being of non‐white ethnicity has been reported as a risk factor for later booking in the general UK population 13, 14, 15, 16, and also in the NSHPC data 17. US data show that women of black ethnicity experience poorer antenatal care usage 18, 19, as does the French Perinatal Cohort 20. This may in part reflect differing cultural beliefs around antenatal care 21, as well as possible barriers to accessing care, including lack of knowledge about the health care system and language difficulties 13, 22. Culturally appropriate promotion of antenatal care services among ethnic minority women in the UK is one approach that may be beneficial 23. Newly diagnosed women born abroad also started ART later than those born in the UK/Ireland (there was no such association among those previously diagnosed), and this was particularly apparent among women from Western Africa. This association persisted in several sensitivity analyses, indicating that it was not simply related to women arriving in the UK late in pregnancy, nor just a reflection of late presentation for antenatal care. In previously diagnosed women, late booking was more common outside London, whereas for newly diagnosed women, late booking was more common in London. Reasons are unclear but may reflect the likely greater concentration of newly arrived migrants in London who may have particular difficulties accessing health care.

Current UK standards state that positive HIV results should be received by maternity services within 10 working days of the sample being taken and the result given to women within 10 days of this, and that there should be ‘urgent’ referral to the HIV specialist team thereafter 24. Although a majority of newly diagnosed women had prompt laboratory assessment, over a third had a gap of ≥ 4 weeks between booking and laboratory assessment. Taking date of laboratory assessment as a proxy for first appointment with the HIV specialist team, the finding suggests that this is an area of practice that may need improving. It is, however, encouraging that, on the whole, newly diagnosed women started ART reasonably promptly with respect to current UK guidelines 4. Those with lower CD4 counts started ART significantly sooner, and those with lower CD4 counts or high viral loads had shorter gaps from laboratory testing to ART initiation. Looking forwards, there is a move towards recommending earlier ART initiation. UK treatment guidelines are being revised, and are likely to recommend ART initiation regardless of CD4 count in light of the results of the Strategic Timing of AntiRetroviral Therapy (START) trial 25; pregnancy guidelines are consequently expected to change. Recently updated US pregnancy guidelines state that ART should be considered as soon as HIV is diagnosed 26.

Higher parity was associated with later booking (most notably among previously diagnosed women), a trend also observed in the general UK population 13, 15, 27. Previously diagnosed women with at least three previous births also started ART later. Reasons are likely to be complex. Different solutions may be required for women who start ART late as a consequence of delayed booking for antenatal care, and those who book for care in good time but experience subsequent delays in ART initiation. An audit of women in the NSHPC who received no antenatal ART or a short duration of ART reported that, among those previously diagnosed, declining treatment was the most common reason (11 of 15; 73%)^28^.

Women with a prior HIV diagnosis, many of whom will have been diagnosed during a previous pregnancy 29, 30, should be receiving ongoing HIV care, which might be expected to facilitate early ART initiation in subsequent pregnancies. However, we have previously demonstrated that retention in HIV care after delivery may be suboptimal, with two‐fifths of women presenting with a second pregnancy not being on treatment despite an immunological indication 31. Women not on ART at conception booked later than those conceiving on treatment, which is of concern as timely booking and prompt referral to HIV services are needed, especially for those with low CD4 counts. Higher levels of loss to follow‐up from HIV care have been reported among people not receiving treatment 32, and disengagement from services may have particularly serious consequences for pregnant women. Although antenatal ART was started earlier in previously diagnosed women with lower CD4 counts, in adjusted analyses there was little difference in ART timing between those with the lowest CD4 counts and the healthiest women. This suggests that there may be a group of high‐risk women who are poorly engaged with care and/or suboptimally managed. It should also be borne in mind that, although women with a CD4 count < 350 cells/μL should be on ART at conception, those who are not may have concerns about initiating ART early in pregnancy because of fears of teratogenicity.

There are some limitations to this analysis. Women cannot book for antenatal care in the UK before they have entered the country. Although the NSHPC collects date of arrival in the UK, this information was completely missing for around one‐third of women born abroad. Even among those with information reported, over half had only year of arrival (not actual date) provided, so it was not appropriate to utilize this variable in the main analyses; instead, the effect of timing of arrival on the findings was assessed in sensitivity analyses. Week of antenatal booking was missing for 9% of women (81% of whom had a prior HIV diagnosis). Although only a small minority of this group did not start ART before delivery (3.3% of those not conceiving on treatment), this was higher than for women who booked at < 13 weeks (1.1%). Some of those with missing booking date may represent a particularly high‐risk group requiring targeted interventions to help them engage with services. Meanwhile, earliest laboratory test date during current pregnancy is likely to be the date on which the sample was taken, although a minority of NSHPC respondents may report other dates, such as the date on which the result was reported from the laboratory. There may have been unadjusted confounding as a result of some variables (e.g. socioeconomic factors) not being available in the NSHPC, which has to limit data items collected to maximize reporting. Where delays in ART initiation were apparent, it was not possible to assess to what extent these were health system‐ or provider‐related, structural (socioeconomic environment), or related to women's individual circumstances, beliefs and choices. Further exploration of this would be helpful, but is beyond the scope of this analysis.

These analyses, based on comprehensive national data, demonstrate significant improvements in the timeliness of antenatal care booking and antenatal ART initiation in the UK in recent years. However, they also reveal some important demographic variations in access to or uptake of care and highlight the fact that some groups of HIV‐positive women continue to face barriers to timely initiation of antenatal care and ART.
